# Environmental stresses induce transgenerationally inheritable survival advantages via germline-to-soma communication in *Caenorhabditis elegans*

**DOI:** 10.1038/ncomms14031

**Published:** 2017-01-09

**Authors:** Saya Kishimoto, Masaharu Uno, Emiko Okabe, Masanori Nono, Eisuke Nishida

**Affiliations:** 1 Department of Cell and Developmental Biology, Graduate School of Biostudies, Kyoto University, Sakyo-ku, Kyoto 606-8502, Japan; 2 AMED-CREST, Japan Agency for Medical Research and Development, 1-7-1 Otemachi, Chiyoda-ku, Tokyo 100-0004, Japan

## Abstract

Hormesis is a biological phenomenon, whereby exposure to low levels of toxic agents or conditions increases organismal viability. It thus represents a beneficial aspect of adaptive responses to harmful environmental stimuli. Here we show that hormesis effects induced in the parental generation can be passed on to the descendants in *Caenorhabditis elegans*. Animals subjected to various stressors during developmental stages exhibit increased resistance to oxidative stress and proteotoxicity. The increased resistance is transmitted to the subsequent generations grown under unstressed conditions through epigenetic alterations. Our analysis reveal that the insulin/insulin-like growth factor (IGF) signalling effector DAF-16/FOXO and the heat-shock factor HSF-1 in the parental somatic cells mediate the formation of epigenetic memory, which is maintained through the histone H3 lysine 4 trimethylase complex in the germline across generations. The elicitation of memory requires the transcription factor SKN-1/Nrf in somatic tissues. We propose that germ-to-soma communication across generations is an essential framework for the transgenerational inheritance of acquired traits, which provides the offspring with survival advantages to deal with environmental perturbation.

## Abstract (web summary)

Environmental stress causes epigenetic changes but it is unclear if such changes are transgenerational. Here, the authors show that in *C. elegans*, increased resistance to oxidative stress and proteotoxicity in the parental generation and linked epigenetic changes are transmitted to subsequent generations.

Epigenetic changes, such as histone post-translational modifications induced by environmental factors, had seemed to reset entirely during gamete formation. Recent studies, however, have shown that some histone marks can persist, even through meiosis, indicating the perpetuation of epigenetic memory^1^. Strikingly, emerging evidence suggests that specific epigenetic memories are transmitted across generations and lead to phenotypic variations in the offspring^2^,^3^,^4^,^5^, although the underlying molecular mechanisms remain to be elucidated.

Hormesis effects, adaptive responses to low-doses storessors, have been reported to improve the functional ability of cells and organisms across a variety of animal species^6^,^7^,^8^,^9^. Such defensive mechanisms against environmental deterioration are crucial to the survival of species in the wild. Non-lethal exposure to stressors in early life can increase the stress resistance of animals and extend the lifespan, suggesting the long-lasting hormesis effects throughout life^10^,^11^,^12^. Recent studies suggest that ancestral environmental conditions can influence the phenotypes of progeny^13^,^14^,^15^,^16^. However, it is unclear whether beneficial hormesis effects can be transmitted to the offspring.

Here we address this question and uncover the transgenerational epigenetic inheritance of hormesis effects in the nematode *C. elegans*. Moreover, our analysis identifies the factors and tissues responsible for the formation, maintenance and elicitation of the epigenetic memory, respectively, and thus reveals the germ-to-soma communications involved in the transgenerational inheritance of acquired traits.

## Results

### Stress exposure enhances oxidative stress resistance

**Figure 1:**
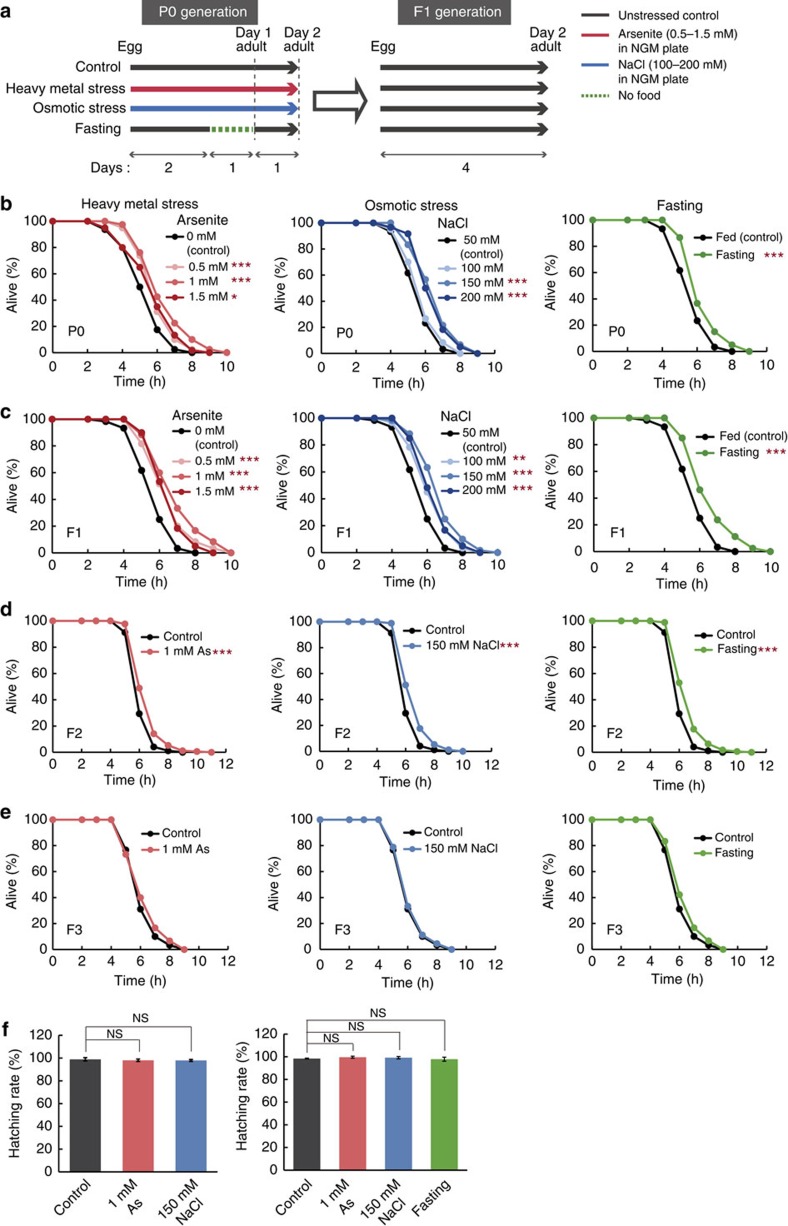
Transgenerational inheritance of increased resistance to oxidative stress. (**a**) Scheme for exposure to environmental stressors. P0 parents were subjected to each stressor during developmental stages as indicated and the F1 descendants were raised under unstressed conditions. (**b**–**e**) Oxidative stress resistance of the stressed P0 parents (**b**), self-fertilized F1 (**c**) on day 2 adulthood in 2.3 mM H_2_O_2_, F2 (**d**) and F3 (**e**) descendants on day 1 adulthood in 1.7 mM H_2_O_2_ compared with control groups. Three independent experiments are integrated into each survival curve. Statistical significance was calculated by log-rank test. *P<0.05, **P<0.01, ***P<0.005. Mean survival time and statistics are presented in Supplementary Table 1. (**f**) Hatching rate of the stressed P0 parents (left) and the F1 (right) descendants. Statistical significance was calculated by Student's *t*-test. Error bars represent the mean±s.d. of three independent experiments. NS, not significant; AS, arsenite.

We first examined whether exposure to environmental stressors only during developmental stages affects the viability of the individuals. We grew hermaphrodites under various stress conditions: heavy metal (arsenite), hyperosmosis (NaCl) and fasting (1 day during the L4 larval stage). Although the animals subjected to these stressors, except for hyperosmosis, showed a slight developmental delay and a shorter body length compared with unstressed control animals at day 2 adulthood, the germline developed completely, and the egg laying started in all of the conditions (Supplementary Fig. 1a). Because there was no difference in stress resistance between day 1 adulthood (young adult) and day 2 adulthood in wild-type animals (Supplementary Fig. 1b), the slight changes in the developmental rate and the body size of surveyed animals may not affect the results of the stress resistance assay. Thus, we performed the stress resistance assay on day 2 adulthood (Fig. 1a). Our measurements showed that each of these environmental stressors during developmental stages led to an increase in animals' resistance in adulthood to a fatal oxidative stressor, hydrogen peroxide (H_2_O_2_) (Fig. 1b). Exposure to fasting and hyperosmosis also increased the resistance to paraquat treatment that causes oxidative stress via the induction of reactive oxygen species (Supplementary Fig. 1c). These results indicate that environmental stressors during developmental stages induce an adaptive, defensive response, that is, hormesis, in adulthood. This hormesis effect can also be interpreted as cross tolerance, in which the animals subjected to primary stressors exhibit the enhanced resistance to another type of stressors.

To investigate the intergenerational effects of parental hormesis, we grew F1 hermaphrodites, which arose from the self-fertilization of stressed P0 generation, under unstressed conditions and measured their stress resistance. We found that F1 descendants from stressed P0 parents showed enhanced resistance to oxidative stress compared with those from control parents (Fig. 1c; Supplementary Fig. 1d). Exposure to heavy metal (arsenite) and hyperosmosis (NaCl) produced a biphasic dose–response effect on stress resistance, and these were the optimal concentrations of arsenite and NaCl on the exposure in the P0 generation; the extent of the increase in stress resistance of the F1 descendants was largest at 1 mM arsenite (21.5%) for the case of heavy metal, and at 150 mM NaCl (18.1%) for the case of hyperosmosis, respectively. The exposure to stressors in the parental generation still had a beneficial influence on F2 generations, although the transgenerational effect gradually declined (Fig. 1d; Supplementary Fig. 1e). After the F3 generations, little or no significant differences in stress resistance were observed between descendants from the stressed P0 parents and those from the unstressed ones (Fig. 1e). Both the stressed P0 parents and the self-fertilized F1 descendants normally hatched and developed (Fig. 1f), suggesting that the stress exposure during developmental stages in the P0 parents did not induce a culling of relatively weak individuals from the experimental groups. Taken together, these results indicate that environmental conditions of the parental generation affect the viability of subsequent generations.

Several studies suggest that enhanced viability leads to a decrease in reproductive ability^17^. To evaluate such a trade-off effect for the increase in stress resistance described above, we measured the brood size, and lifespan of stressed P0 parents and their F1 descendants. We found that P0 hermaphrodites exposed to heavy metals showed a small decrease in progeny numbers, whereas the reproductive ability of animals subjected to hyperosmosis or fasting was comparable to that of the control group (Supplementary Fig. 2a). In addition, stressor exposures in the parental generation had no significant effect on fecundity of the F1 descendants (Supplementary Fig. 2a). Similar results were observed in the lifespan measurements; P0 parents subjected to each stressor tended to exhibit a slightly shortened lifespan compared with the control group, but the F1 descendants did not display a shortened lifespan (Supplementary Fig. 2b,c). These results are not necessarily consistent with the trade-off mechanism, but rather suggest that environmental stresses during the developmental stages may have both beneficial and harmful effects on organismal phenotypes, especially on the stressed parental generation.

### Stress exposure enhances resistance to proteotoxicity

**Figure 2:**
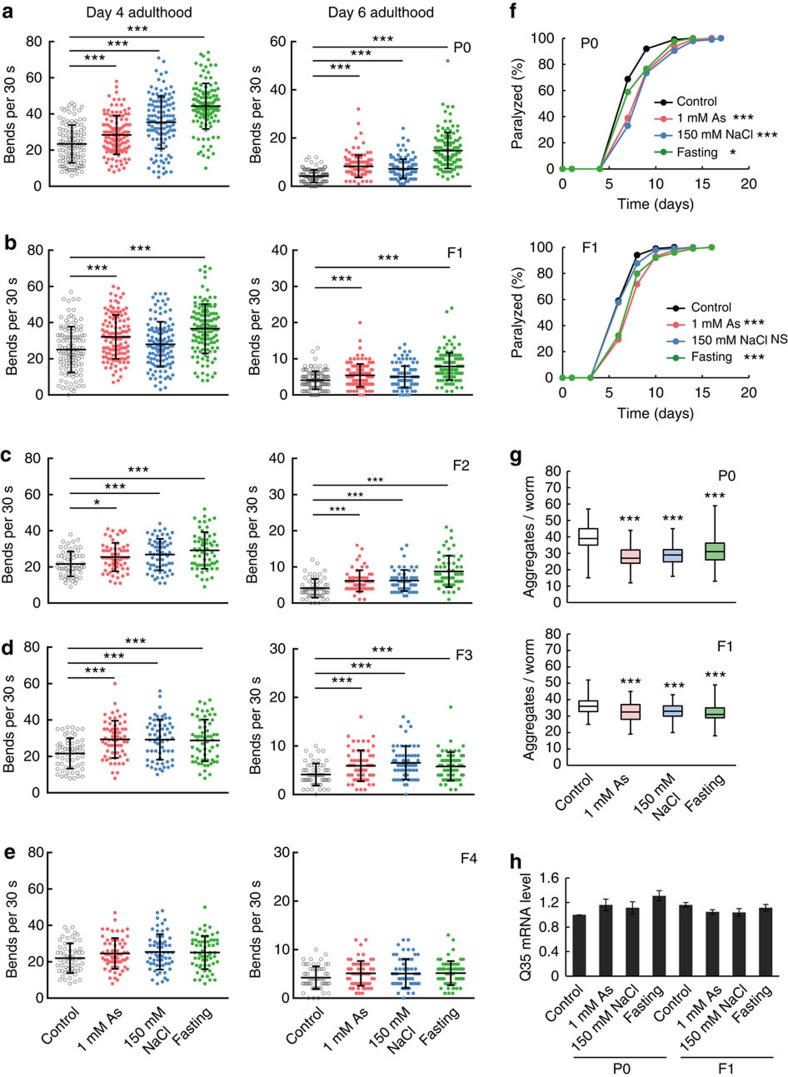
Transgenerational inheritance of increased resistance to proteotoxicity. (**a**–**e**) Thrashing rate of the stress-exposed P0 transgenic animals expressing polyQ (**a**), self-fertilized F1 (**b**), F2 (**c**), F3 (**d**) and F4 (**e**) descendants compared with control groups (left, day 4 adulthood; right, day 6 adulthood). Each point represents the number of the body bend of a single animal over a period of 30 s. Two or three independent experiments are integrated into each scatter graph (*n*=120 for **a** and **b**, *n*=60 for **c**–**e**). Mean±s.d., *P<0.05, **P<0.01, ***P<0.005, Mann–Whitney *U* test. (**f**) Rate of paralysis of the stressed P0 (upper) and the self-fertilized F1 (lower) polyQ animals. Representative data of two independent experiments are shown. The sample size and *P* values are presented in Supplementary Table 2. NS, not statistically significant; *P<0.05, ***P<0.005, log-rank test. (**g**) Number of polyQ protein aggregates in the stressed P0 (upper) and the F1 (lower) on day 4 adulthood. Two independent experiments are integrated into each graph (*n*=100). Box plots represent median and interquartile ranges, and whiskers represent minimum to maximum values. ***P<0.001, Mann–Whitney *U* test. (**h**) Relative expression levels of Q35::YFP transgene. Statistical significance was calculated by Student's *t*-test. Error bars represent the mean±s.e.m. of three independent experiments. No statistical significance was observed in each sample compared with the P0 control.

To further investigate inheritable traits acquired in the P0 generation, we focused on a resistance to proteotoxity. We evaluated the motility phenotype of a transgenic animal expressing polyglutamine (polyQ) fused to the yellow fluorescent protein (YFP) in the body wall muscle (Punc-54::Q35::YFP), which displays age-dependent polyQ aggregation leading to motility defects^18^. Animals, which were subjected to various stressors until day 2 adulthood as described above (Fig. 1a), exhibited a markedly more rapid-thrashing movement than unstressed animals (Fig. 2a). Remarkably, the self-fertilized F1 descendants, and even F2 and F3 descendants from stressed P0 parents also showed a more rapid-thrashing movement than the descendants from unstressed parents (Fig. 2b–d). F4 descendants also showed a slightly more rapid movement than the control group, but there was no statistical significance in the differences (Fig. 2e). To confirm these observations, we additionally examined an age-dependent paralysis phenotype of the polyQ-expressing strains. The results showed that the rates of paralysis in the both stressed P0 parents and the F1 descendants are alleviated compared with those in the unstressed control groups (Fig. 2f), suggesting that environmental stress exposure induces the increase in the resistance to proteotoxicity. In addition, parental stress exposure resulted in the reduction of polyQ aggregates in both generations (Fig. 2g). To address a possibility that the stress conditions may simply downregulate transcription of the polyQ transgene, we examined the polyQ messenger RNA expression levels and found that there was no significant difference in the transcription level between stressed and unstressed animals (Fig. 2h). Collectively, these results demonstrate that stress exposure during the developmental stages in the P0 generation improves proteostasis of the individuals and mitigates the aggregation of aberrant proteins, leading to protective effects against proteotoxity, which are remarkably transmitted to the subsequent generations.

### Paternal and maternal inheritance of hormesis effects

**Figure 3:**
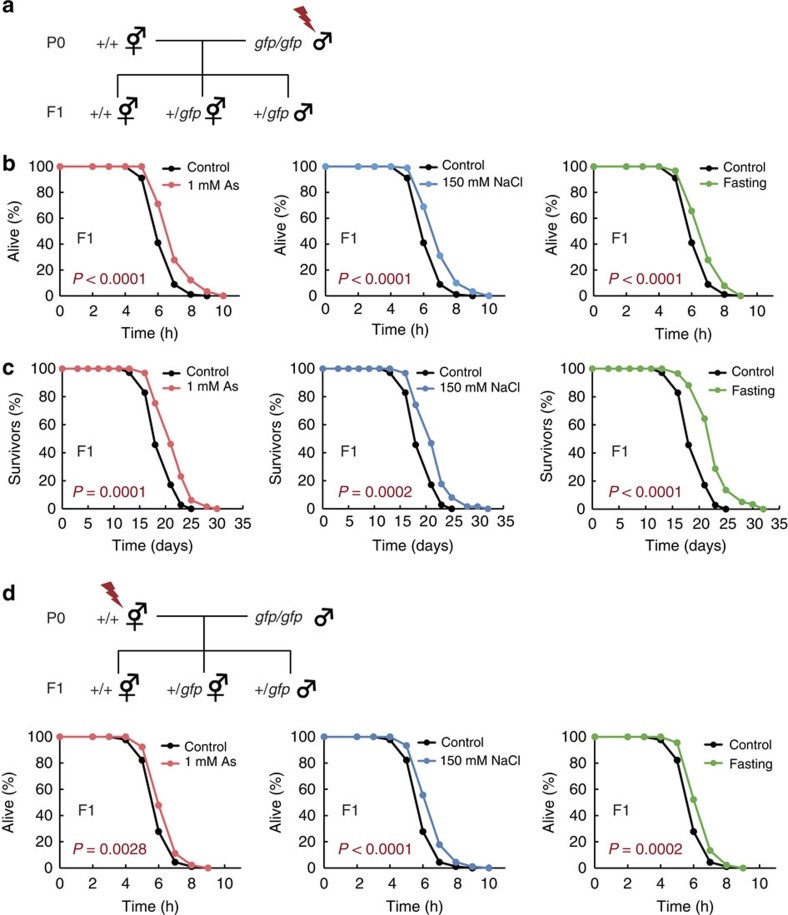
Paternal and maternal inheritance of hormesis effects. (**a**) Scheme for isolation of the crossed F1 descendants whose male parents are subjected to stressors. GFP expression allows us to distinguish the crossed F1 descendants from the self-fertilized ones. (**b**) Oxidative stress resistance (2.3 mM H_2_O_2_) of the crossed F1 descendants on day 1 adulthood compared with control groups. Three independent experiments are integrated into each survival curve (*n*=90). (**c**) Lifespan of the crossed F1 descendants. Representative data of seven independent experiments are shown. (**d**) Scheme for isolation of the crossed F1 descendants whose hermaphrodite parents are subjected to stressors (upper). Oxidative stress resistance (2.3 mM H_2_O_2_) of the crossed F1 descendants on day 1 adulthood compared with control groups (lower). Three independent experiments are integrated into each survival curve (*n*=90). Mean lifespan and statistics are presented in Supplementary Tables 1 and 3. *P* values were calculated by log-rank test.

Multiple studies have demonstrated that adverse maternal experiences affect metabolic phenotypes in the offspring^19^, although the intergenerational influences seem to include the contribution of parental effects, such as maternally deposited cytoplasmic elements or direct exposure of the gamete to the stressors *in utero*^3^,^4^,^5^. Our findings show that hormesis effects induced by environmental stressors in the P0 generation can be transmitted across generations; however, the extent to which the parental effects would contribute to the phenotypic outcome in the offspring has to be determined. To address this question, we exposed males to various stressors, crossed the stressed parental males with unstressed hermaphrodites and examined the stress resistance of the F1 descendants (Fig. 3a). As male parents harbour the transgene-encoding P*myo-2*::*gfp*, the F1 descendants generated by crossing exhibit GFP expression, which enables us to distinguish the crossed descendants from self-fertilized ones. The results showed that exposure of only male parents to stressors during the developmental stages resulted in the increased oxidative stress resistance in the F1 descendants (Fig. 3b). Remarkably, these F1 descendants exhibited lifespan extension (Fig. 3c). This extended lifespan was also observed in the F1 descendants derived from parents expressing another type of transgene (Supplementary Fig. 3). These results suggest that these animals may improve the fundamental physiological properties without stress stimuli as a trigger. Then, we asked whether the transmission of hormesis effects to the next generation would be regulated only through a sperm-dependent pathway. To address this question, we crossed stressed parental hermaphrodites with unstressed males and selected the F1 descendants displaying GFP expression. As a result, we found that the F1 descendants, whose parental hermaphrodites were subjected to the stressor exposure showed the increased stress resistance compared with control descendants from unstressed parents (Fig. 3d), suggesting that the hormesis effects could be transmitted across generations either paternally or maternally. These observations strongly suggest that the transgenerational inheritance of hormesis effects would be passed on to subsequent generations by certain epigenetic information carriers rather than just through simple maternal effects.

### Transgenerational inheritance requires histone modifiers

**Figure 4:**
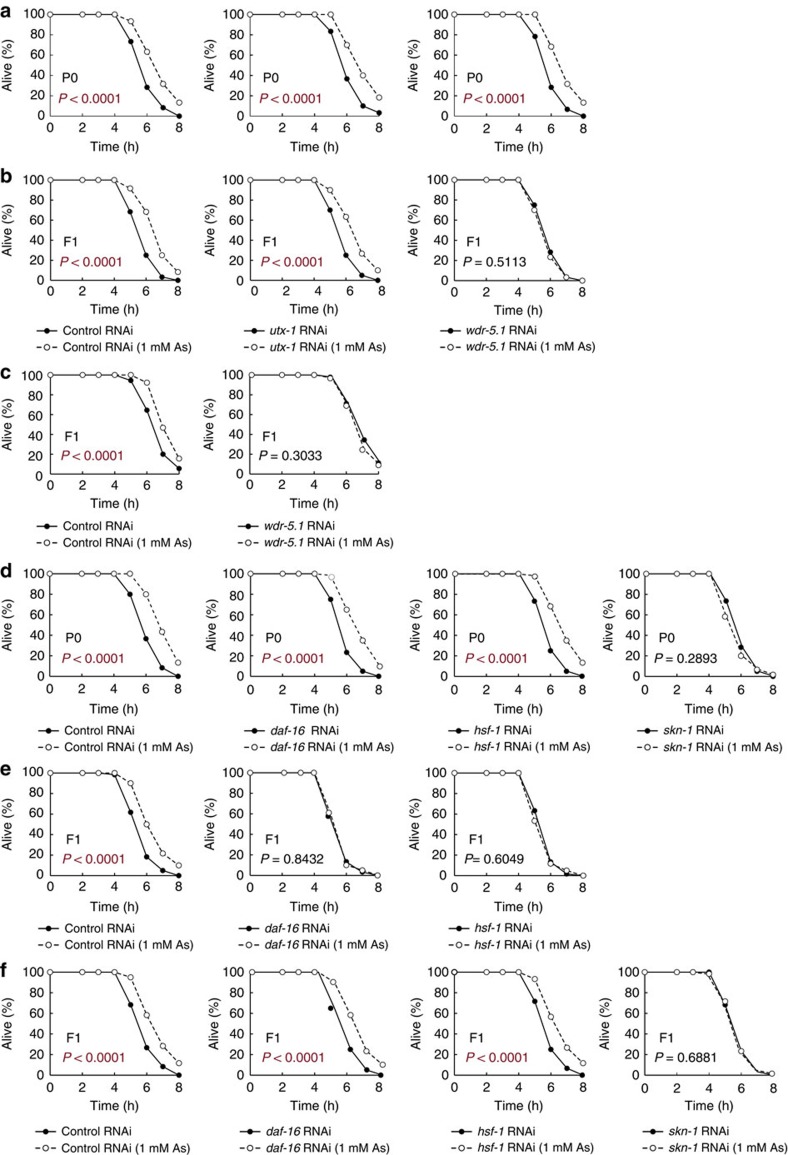
The H3K4me3 complex and two transcription factors are required for the transgenerational inheritance. (**a**,**b**) Oxidative stress resistance (1.7 mM H_2_O_2_) of the stressed P0 parents treated with RNAi (left, empty vector (control); middle, *utx-1*; right, *wdr-5.1*) on day 2 adulthood (**a**) and the self-fertilized F1 descendants on day 1 adulthood (**b**) compared with control groups. Two independent experiments are integrated into each survival curve (*n*=60). (**c**) *wdr-5.1* RNAi treatment in F1 descendants from the stressed P0 parents leads to the suppression of the increase in the oxidative stress resistance. Three independent experiments are integrated into each survival curve (*n*=90). (**d**,**e**) Oxidative stress resistance (1.7 mM H_2_O_2_) of the stressed P0 parents on day 2 adulthood treated with RNAi of indicated genes (**d**) and the self-fertilized F1 descendants on day 1 adulthood (**e**) compared with control groups. (**f**) *daf-16* or *hsf-1* RNAi treatment performed in the F1 descendants does not compromise the increased resistance, whereas *skn-1* RNAi leads to the suppression of the increase in the oxidative stress resistance. Two independent experiments are integrated into each survival curve (*n*=60). *P* values were calculated by log-rank test.

Epigenetic alterations, involving DNA methylation, histone post-translational modification and chromatin remodelling, have been described as one of the hallmarks of ageing^20^,^21^. It has been reported that genetic manipulations of histone-modifying enzymes can alter specific histone mark levels and in turn affect the lifespan of individuals. For example, the deficiency in the histone H3K4me3 regulatory complex composed of ASH-2, WDR-5 and SET-2 leads to lifespan extension^22^, which can be inherited until the third generation^23^, and the reduction in the histone H3K27me3 demethylase UTX-1 also extends lifespan in *C. elegans*^24^,^25^. Given that the recent findings suggest the involvement of epigenetic modifications in the transgenerational inheritance of parental phenotypes^3^,^4^,^5^, we hypothesized that exposure to stressors in the parental generation induces epigenetic alterations, which would produce an enhanced viability and could be maintained and transmitted to the next generations. To test this hypothesis, during heavy metal stressor exposure in the P0 generation, we inhibited some histone modifiers that are related to longevity and evaluated the stress resistance of the objective parents and offspring. Unexpectedly, knockdown of genes encoding the H3K27me3 demethylase *utx-1* or the H3K4me3 complex components (*wdr-5.1*, *ash-2* and *set-2*) in the P0 generations had no effect on the increased stress resistance of the parents (Fig. 4a; Supplementary Fig. 4a), suggesting that parents acquire stress-induced hormesis effects independently of these histone modifiers. However, we observed that knockdown of the H3K4me3 complex components apparently failed to increase the resistance of F1 descendants (Fig. 4b; Supplementary Fig. 4b). Consistent with the above results, animals carrying the *wdr-5.1 (ok1417)* or *set-2 (ok952)* mutation showed a stressor exposure-induced increase in the stress resistance of the P0 generation, but did not show increased resistance in the F1 generation (Supplementary Fig. 4c). Given that knockdown of *utx-1* or H3K4me3 demethylase *rbr-2* did not affect the resistance of descendants (Fig. 4b; Supplementary Fig. 4d), the inheritance of hormesis effects seems to require specific histone modification factors, such as H3K4 trimethylation complex or possibly additional epigenetic mediators. In these experiments, gene knockdown effects induced by feeding RNA interference (RNAi) persisted beyond generations (Supplementary Fig. 4e,f). So, to gain further insight into the time period in which the H3K4me3 complex functions, we knocked down components of the complex only in the F1 generation, which was derived from the stressed P0 generation. The results showed that *wdr-5.1* knockdown in F1 descendants led to the suppression of the increase in resistance (Fig. 4c) and thus suggest that the H3K4me3 complex functions at least in the subsequent generation and is required for the maintenance of epigenetic marks. These findings demonstrate that the histone H3K4me3 complex plays an essential role in accomplishing a transmission of stress-induced hormesis effects from one generation to the next. However, there were no significant differences in the global H3K4me3 levels between stressed and unstressed animals (Supplementary Fig. 5). One possibility is that epigenetic alterations might occur in certain specific gene loci or specific tissues, but cannot be detected by Western blotting analysis of whole-body samples.

### Transcription factors mediate heritable memories

To examine the molecular mechanisms underlying the induction of heritable hormesis effects, we evaluated the contribution of three well-characterized transcription factors that play an important role in intrinsic stress responses under typical stress conditions^26^. DAF-16 is a forkhead transcriptional factor, a key downstream effector of the insulin/insulin-like growth factor (IGF) signalling pathway, and is the most studied modulator of lifespan. Heat-shock factor-1 (HSF-1) is a major transcriptional activator of heat-shock protein genes that function in stress response and maintain proteostasis. SKN-1, the nematode homologue of NRF2, orchestrates a well-conserved oxidative stress response. First, we knocked down each of the three pivotal transcriptional factors in the P0 generation that was subjected to heavy metal exposure during the developmental stages. Knockdown of either *daf-16* or *hsf-1* did not compromise the increase in resistance to oxidative stress in the P0 generation (Fig. 4d). On the other hand, knockdown of *skn-1* abolished the increased resistance (Fig. 4d), which seems plausible given the central function of SKN-1 in the oxidative stress response. Because knockdown of *skn-1* in the P0 generation induced the embryonic lethal phenotype of the offspring, we could not assay the stress resistance of the F1 descendants. Interestingly, knockdown of either *daf-16* or *hsf-1* led to the suppression of the increase in the stress resistance of F1 descendants (Fig. 4e), suggesting that these two transcription factors are required for the transgenerational inheritance of hormesis effects. Under the conditions used, the RNAi-induced gene knockdown effects persisted in the next generation (Supplementary Fig. 4e,f). Then, to address the question of when DAF-16 and HSF-1 are required, we knocked down these factors only in the F1 descendants derived from the stressed P0 parents. The results showed that knockdown of either *daf-16* or *hsf-1* in the F1 generation did not result in the suppression of the increase in the stress resistance, whereas knockdown of *skn-1* abolished the increase in the resistance as in the case of the P0 parents (Fig.). These results demonstrate that DAF-16 and HSF-1 work in the P0 generation to transmit the acquired trait of the increased stress resistance induced by environmental stressors to the descendants, whereas SKN-1 is required for the execution of the oxidative stress response. Similar results were obtained from the experiments using genetic mutants of *daf-16*, *hsf-1* and *skn-1* (Supplementary Fig. 6), yet the mutation of *daf-16* or *hsf-1* seems to partially suppress the increased stress resistance in the P0 generation, indicating that DAF-16 and HSF-1 may in part contribute to the stress response in the P0 parents. Accordingly, DAF-16 and HSF-1 should mainly function to transmit the heritable memories that induce hormesis effects, and SKN-1 may work to execute the hormesis effects *per se*. Because DAF-16 and HSF-1 have been reported to cooperate with each other to regulate lifespan and stress resistance^27^, it is possible that these factors also act in concert to mediate the transgenerational inheritance of hormesis effects.

### Germ-to-soma communications in transgenerational inheritance

**Figure 5:**
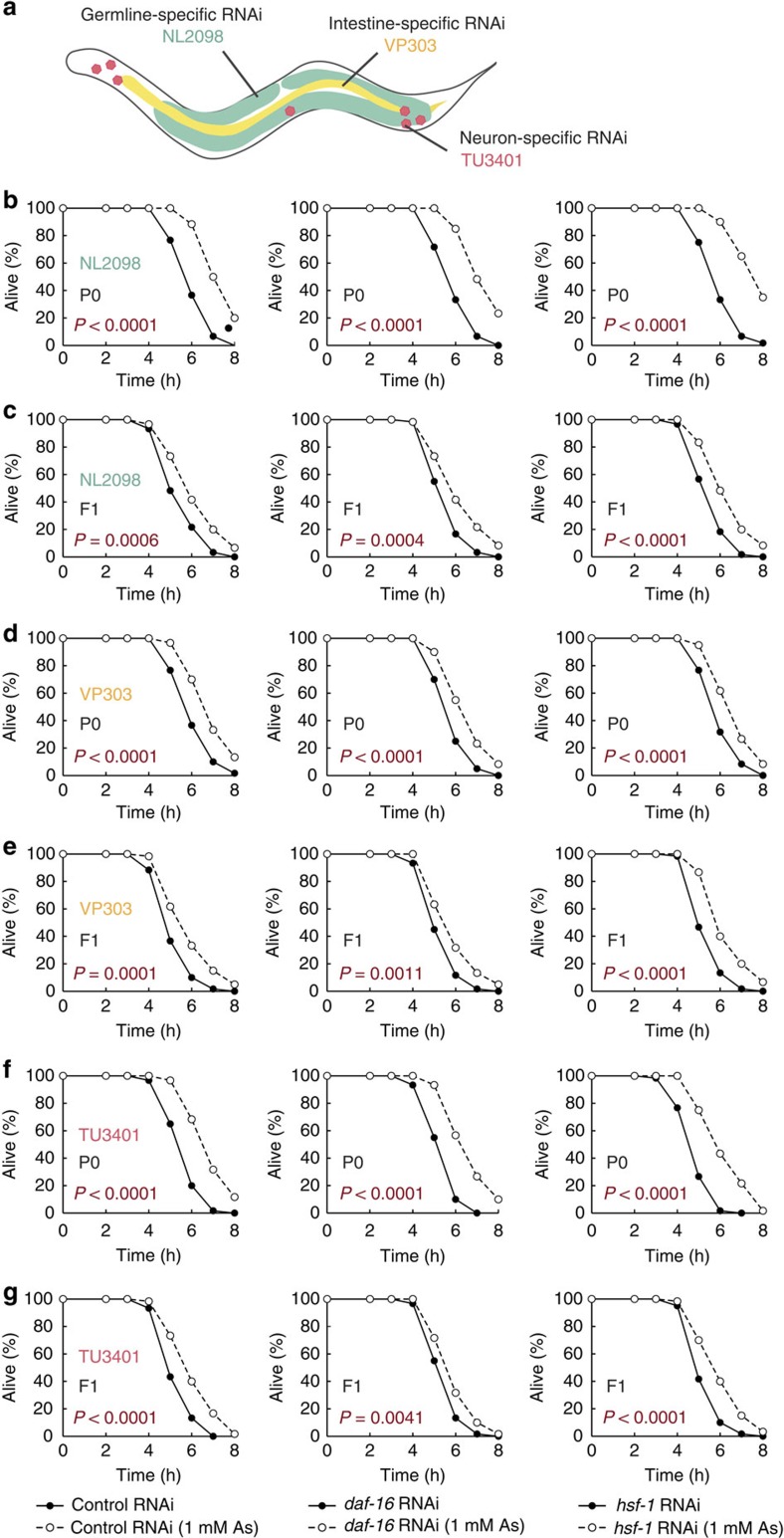
Tissue-specific RNAi of *daf-16* or *hsf-1*. (**a**) Strains used for the tissue-specific gene knockdown. (**b**,**c**) Oxidative stress resistance (2 mM H_2_O_2_) of the stressed P0 parents treated with germline-specific RNAi (left, empty vector (control); middle, *daf-16*; right, *hsf-1*) on day 2 adulthood (**b**) and the self-fertilized F1 descendants on day 1 adulthood (**c**). (**d**,**e**) Oxidative stress resistance (2 mM H_2_O_2_) of the stressed P0 parents treated with intestine-specific RNAi on day 2 adulthood (**d**) and the self-fertilized F1 descendants on day 1 adulthood (**e**). (**f**,**g**) Oxidative stress resistance (2 mM H_2_O_2_) of the stressed P0 parents treated with neuron-specific RNAi on day 2 adulthood (**f**) and the self-fertilized F1 descendants on day 1 adulthood (**g**). Two independent experiments are integrated into each survival curve (*n*=60). *P* values were calculated by log-rank test.

To determine tissues in which the transcription factors and the histone modifier function, we performed tissue-specific gene knockdown experiments by using several *C. elegans* strains that are able to process RNAi efficiently only in particular tissues, such as germline, intestine and neuron, but not in other tissue^28^ (Fig. 5a). First, we performed the tissue-specific RNAi of *daf-16* and *hsf-1* in the germline of P0 generation that was subjected to heavy metal exposure. Similar to the case of the systemic RNAi described in Fig. 4, knockdown of either *daf-16* or *hsf-1* in the germline did not compromise the increase in resistance to oxidative stress in the P0 generation (Fig. 5b). However, the descendants exhibited the increase in the stress resistance, contrary to the suppression of the resistance in the case of the systemic RNAi (Fig. 5c); therefore, DAF-16 and HSF-1 in somatic tissues of the P0 generation are sufficient for the formation of the epigenetic memory that induces the viability in the descendants. Then, we performed intestine-specific and neuron-specific RNAi of *daf-16* and *hsf-1*. However, no significant decline in the increased resistance in each generation was observed in each case (Fig. 5d–g). Thus, the function of DAF-16 and HSF-1 in several tissues may be redundant in the P0 generation; or, these transcription factors act in a certain somatic tissue other than intestine and neuron.

**Figure 6:**
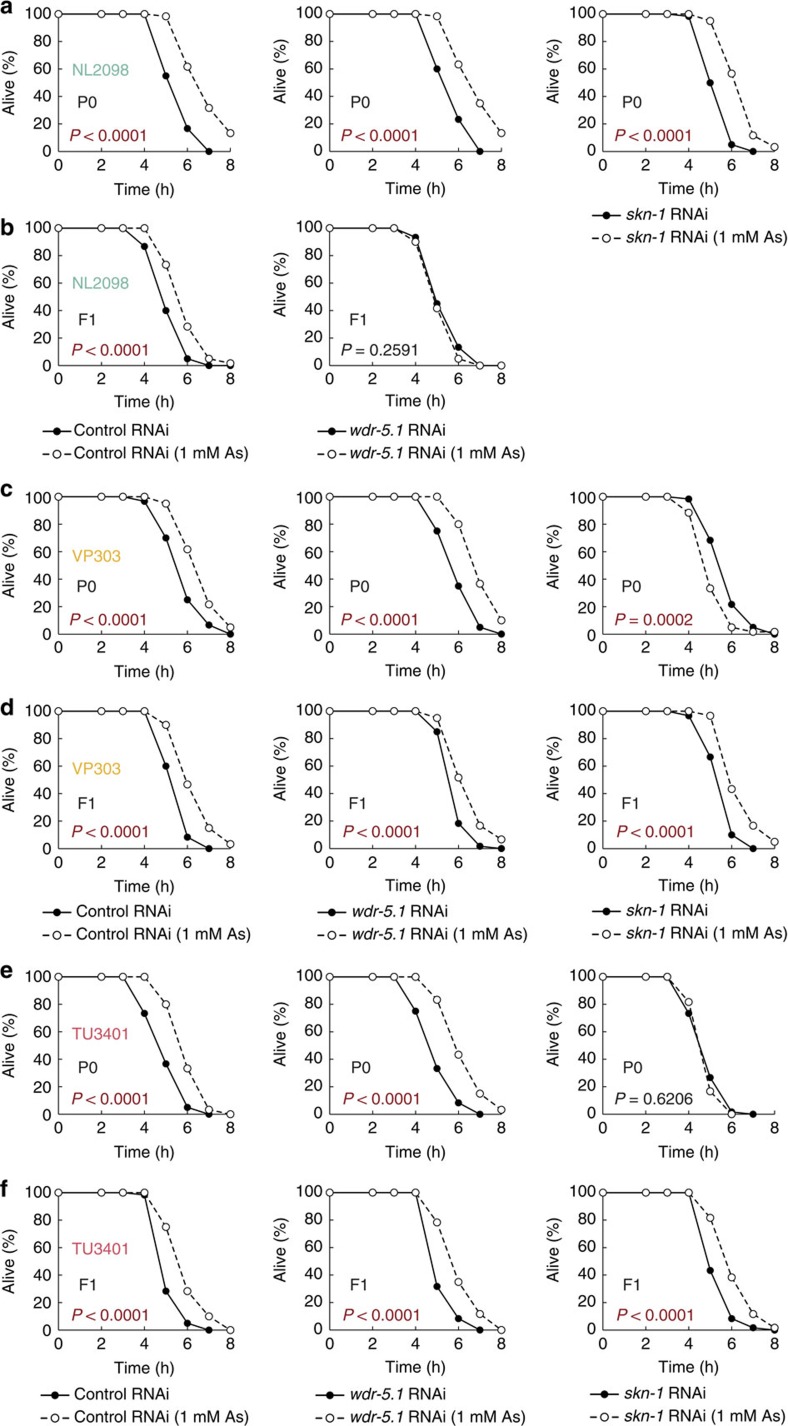
Tissue-specific RNAi of *wdr-5.1* or *skn-1*. (**a**,**b**) Oxidative stress resistance (2 mM H_2_O_2_) of the stressed P0 parents treated with germline-specific RNAi (left, empty vector (control); middle, *wdr-5.1*; right, *skn-1*) on day 2 adulthood (**a**) and the self-fertilized F1 descendants on day 1 adulthood (**b**). (**c**,**d**) Oxidative stress resistance (2 mM H_2_O_2_) of the stressed P0 parents treated with intestine-specific RNAi on day 2 adulthood (**c**) and the self-fertilized F1 descendants on day 1 adulthood (**d**). (**e**,**f**) Oxidative stress resistance (2 mM H_2_O_2_) of the stressed P0 parents treated with neuron-specific RNAi on day 2 adulthood (**e**) and the self-fertilized F1 descendants on day 1 adulthood (**f**). Two independent experiments are integrated into each survival curve (*n*=60). *P* values were calculated by log-rank test.

We then performed tissue-specific knockdown of *wdr-5.1*. Germline-specific RNAi of *wdr-5.1* in the P0 generation led to the suppression of the increase in resistance of the descendants, while keeping the increased resistance in the P0 generation (Fig. 6a,b). RNAi of *wdr-5.1* in intestinal or neuronal tissues, however, had no marked effect on the increased resistance (Fig. 6c–f). Importantly, tissue-specific RNAi in the F1 generation showed that WDR-5 also functions in the germline of the F1 generation (Supplementary Fig. 7a–c). These results indicate that H3K4me3 complex acts in the germline through generations to form, maintain and transmit the epigenetic memory for the phenotype of enhanced resistance to the next generation.

We then performed tissue-specific RNAi of *skn-1*. Intestine-specific and neuron-specific RNAi, but not germline-specific RNAi, of *skn-1* abolished the increased resistance in the P0 generation (Fig. 6a,c,e). Different from *skn-1* RNAi in the whole body or in the germline, *skn-1* RNAi in the somatic tissues did not induce sterility, and the descendants showed the increased resistance (Fig. 6d,f). In the case of somatic tissue-specific RNAi, the knockdown effects may not persist beyond generations, and hence we performed tissue-specific RNAi of *skn-1* in the F1 generation so as to examine whether SKN-1 functions in the offspring. The results showed that *skn-1* RNAi in intestinal and neuronal tissues in the F1 generation abolished the increase in the resistance (Supplementary Fig. 7a–c). Accordingly, we conclude that the function of SKN-1 in somatic tissues, especially in intestine and neurons, is required for the increased stress resistance in each generation.

The tissue-specific RNAi studies reveal that transgenerational inheritance of the increased resistance requires the function of DAF-16 and HSF-1 in parental somatic cells, and that of WDR-5 in the germline through generations, and that the function of SKN-1 in intestinal and neuronal tissues is required for the increased resistance *per se*. These findings suggest that germline-to-soma communications across generations may coordinately regulate transgenerational inheritance of environmental stress-induced hormesis effects.

## Discussion

**Figure 7:**
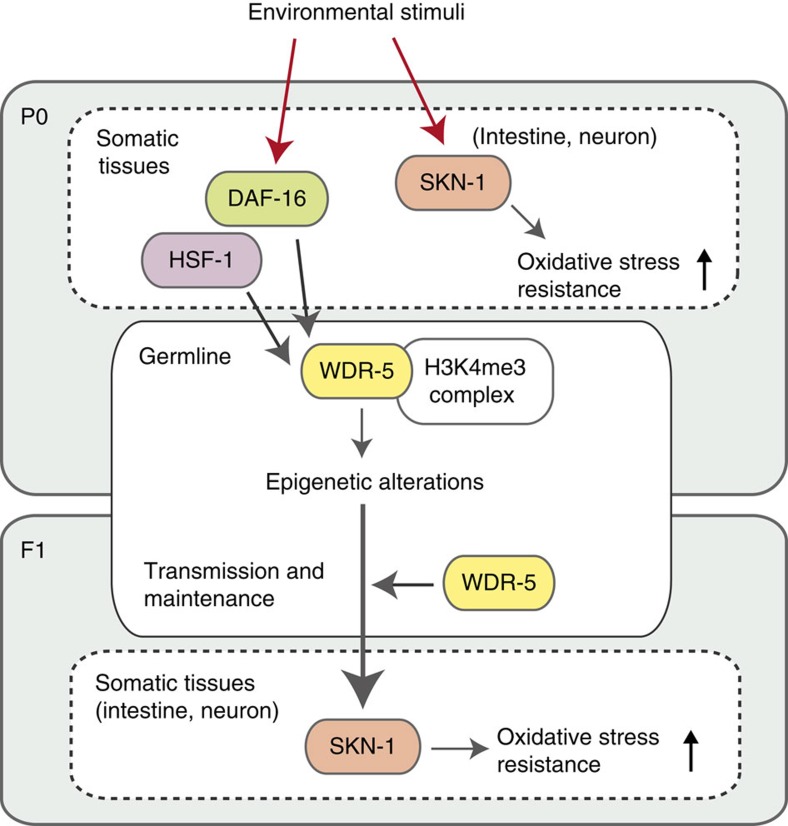
Overview model of transgenerational inheritance of epigenetic memory in response to environmental stimuli. In the P0 generation, somatic DAF-16 and HSF-1 communicate with the H3K4me3 complex in the germline so as to regulate and maintain epigenetic alterations associated with inheritable acquired traits, and the H3K4me3 complex in the filial germline then transfers hormetic information to SKN-1 of the somatic tissues.

Here we have found that stress exposures during developmental stages can induce beneficial effects, which influence phenotypic outcomes in subsequent generations (see Fig. 7 for an overview model). Our findings are in agreement with the previous work of Tauffenberger and Parker^15^, in which they demonstrated that high dietary glucose in the P0 generation induces hormesis effects in the unstressed next generation, and that H3K4me3 modifiers and the insulin/IGF-like signalling components are involved in the underlying mechanisms. Our study newly revealed that exposure of P0 parents to various stressors induces similar hormesis effects in the subsequent generations, indicating the generality of the transgenerational inheritance of acquired traits. In addition, our study demonstrated the paternal transmission, suggesting that the transgenerational inheritance is regulated by certain epigenetic memories. Moreover, we have uncovered the germline-to-soma communication mechanisms by examining, where several identified factors involved in the transgenerational inheritance do function and how they communicate with each other. The function of DAF-16 and HSF-1 are required for the transmission of the enhanced stress resistance to the next generation. SKN-1 is required to increase resistance against oxidative stress in either parents or descendants, which is consistent with the well-established role of SKN-1 as a master regulator of the oxidative stress response. On the other hand, the WDR-5/ASH-2/SET-2 H3K4me3 regulatory complex in the germline is necessary for the expression of persistent phenotypes in the offspring. These findings implicate that somatic DAF-16 and HSF-1 communicate with the H3K4me3 complex in the germline so as to regulate and maintain epigenetic alterations associated with inheritable acquired traits, and the H3K4me3 complex in the filial germline then transfers hormetic information to SKN-1 of the somatic cells, possibly through some mediators such as secreted factors. Further studies will be required to identify the epigenetic alterations at specific loci that are responsible for inheritable hormesis effects. In addition, it is still unclear how DAF-16 and HSF-1 induce epigenetic alterations in response to environmental stressors. A recent report suggests that DAF-16 regulates small RNA-mediated genome silencing^29^, which raises the possibility that DAF-16 and HSF-1 would alter the profile of small RNAs transmitted across generations as information carriers of the hormesis effects. In summary, our finding of the transmission of hormesis effects across generations and the identification of factors and germline-to-soma communication mechanisms involved in the transmission provide a framework for further understanding the molecular mechanisms underlying the transgenerational inheritance of acquired traits. Our study also uncovers an intrinsic strategy in which parental experience during developmental stages form transmissible epigenetic memories, providing survival advantages that elicit enhanced robustness and viability in their descendants.

## Methods

### *C. elegans* strains

All nematodes were cultured using standard *C. elegans* methods^30^. All experiments were performed at 20 °C. The following strains were used in this study: N2: wild-type, AM140: *rmIs132[Punc-54::Q35::yfp]*, RB1304: *wdr-5.1(ok1417)*, RB1025: *set-2(ok952)*, AZ217: *ruIs37[Pmyo-2::gfp;unc-119(+)]*, SJ4100: *zcIs13[Phsp-6::gfp]*, SJ4058: *zcIs9[Phsp-60::gfp]*, SJ4005: *zcIs4[Phsp-4::gfp]*, NL2098: *rrf-1 (pk1417)*, VP303: *rde-1 (ne219); kbIs7[Pnhx-2::rde-1+rol-6 (su1006)]*, TU3401: *sid-1 (pk3321); uIs69[pCF90 (Pmyo-2::mCherry)+Punc-119::sid-1]*, CF1038: *daf-16 (mu86)*, PS3551: *hsf-1 (sy441)*, EU31: *skn-1 (zu135); nT1[unc(n754dm) let]*.

### Environmental stressors conditions

Synchronized eggs were obtained using the bleaching method^31^. Using these synchronous eggs, animals were raised under indicated stress conditions for 4 days (4-day-old animals were defined as day 2 adulthood). For heavy metal or hyperosmosis conditions, animals were raised on normal growth media (NGM) plates containing arsenite (0.5, 1, or 1.5 mM) or high salt growth media plates (100, 150 or 200 mM NaCl), respectively. For starvation or fasting, L4 stage animals raised under normal conditions were transferred to a new NGM plate without *Escherichia coli* OP50 and incubated for 1 day, and then the animals were transferred to NGM plates seeded with OP50. To obtain animals of the F1 generation, 10–20 gravid day 2 adults, which were stressed or unstressed, were transferred onto new NGM plates without environmental stressors, and the animals were allowed to lay eggs for several hours. The parents were then removed, and the plates were incubated at 20 °C for 4 days. This process was repeated to obtain the F2, F3 and F4 generations, though the F1, F2 and F3 generations were not stressed at all.

### Oxidative stress assays

For oxidative stress assays of the P0 generation, animals were raised under indicated environmental stress conditions. For those of the following generations (F1–4), animals were raised without environmental stressors. Two adults at day 1 or 2 were transferred into each well (60-well plate, Greiner bio-one) containing 20 μl of the M9 buffer containing pro-oxidant (1.7 or 2.3 mM hydrogen peroxide (Santoku Chemical Industries Co., Ltd) or 250 mM paraquat (Nacalai Tesque)). Fifteen replicates per condition were assayed. The plates were monitored almost every hour to document the number of animals alive, dead or censored. Animals were scored as dead if they failed to respond to touch with a picker. Summaries of stress resistance experiments are presented in Supplementary Tables 1 and 4.

### Bending and paralysis assays

The bending assay was performed by the following steps based on a previous report^18^. For bending assays of the P0 generation, Q35::YFP-expressing animals were raised under the indicated environmental stress conditions. For those of the following generations, animals were raised without environmental stressors. Synchronous day 2 adults were transferred to 5′ fluoro-2′ deoxyuridine (FUdR; LKT Laboratories)-containing NGM plates seeded with ultraviolet-treated OP50. Day 4 or 6 adults were transferred to a drop of 20 μl of M9 buffer in the 60 well, one worm per single well. Thirty seconds after the transfer, the number of thrashes performed by the worm was counted for 30 s. A thrash was defined as a change in direction of the bend at the midbody of an animal. For the paralysis assay, animals were scored as paralyzed if they were unable to move forward in response to prodding by picker. Animals were transferred every 2 or 3 days to new plates and were marked as paralyzed or not in a blinded manner.

### Aggregates quantification

For the quantification of polyQ aggregates in the P0 generation, Q35::YFP-expressing animals were raised under the indicated environmental stress conditions for 4 days, and then were transferred to normal NGM plates. For the F1 generation, animals were raised without environmental stressors. The fluorescence microscope was used to image animals at day 4 adulthood, and the number of polyQ aggregates was counted (50 animals per each condition, *n*=2). On the basis of a previous study^32^, aggregates were defined as discrete YFP fluorescent structures.

### RNA interference

RNAi was performed by the feeding method as described^33^. Some of the RNAi clones (*wdr-5.1, daf-16, skn-1,* and *utx-1*) were constructed using PCR amplification with the following sets of primers: *wdr-5.1* Fw: 5′-CCCACAATCATCGCTCGTTG-3′, *wdr-5.1* Rv: 5′-CATCCGAGCGCCATATATGA-3′, *daf-16* Fw: 5′-CGGGATCCCGAATTCAGAATGAAGGAGCC-3′, *daf-16* Rv: 5′-TTGCGGCCGCAATTGAAGTTAGTGCTTGGC-3′, *skn-1* Fw: 5′-GGAATTCACGGACAGCAATAATAGG-3′, *skn-1* Rv: 5′-GGAATTCTTCTCTTGAAACATCCTCG-3′, *utx-1* Fw: 5′-TCTGTTCAAACTTGCCGTCAACG-3′, *utx-1* Rv: 5′-GCCTGAAGAGCATCAATGGG-3′.

Other RNAi clones (*set-2, ash-2, rbr-2* and *hsf-1*) were obtained from *C. elegans* RNAi library (Source BioScience).

### Lifespan assays

For lifespan assays of the P0 generation, animals were raised under indicated environmental stress conditions for 4 days. For those of the F1 generation, animals were raised without environmental stressors for 4 days. Approximately 60 animals were transferred to FUdR-containing NGM plates seeded with ultraviolet-treated OP50. The day transferred to FUdR-containing NGM plates was defined as *t*=1 day. We scored death events every other day. Animals were scored as dead if they fail to respond to touch by picker.

### Body length

Day 2 adults of each stress condition in the P0 generation and day 1 adults of each stress condition in the F1 generation were observed with a SZX16 stereomicroscope (Olympus) equipped with a DP73 digital camera (Olympus). The lengths of the animals (30 animals per each condition) were measured using cellSens Standard (Olympus).

### Hatching rate and brood size

For hatching rate assays of the P0 generation, gravid animals were transferred onto NGM plates containing arsenite or high salt growth media and the animals were allowed to lay eggs for several hours. The animals were removed and the number of eggs was counted. The eggs were incubated for 4 days and the number of live animals was counted. For those of the F1 generation, gravid day 2 adults derived from the P0 of each stress condition were transferred onto NGM plates and the animals were allowed to lay eggs for several hours. The animals were removed and the number of eggs was counted. The eggs were incubated for 3 days and the number of live animals was counted. The hatching rate was defined as the number of live animals per the number of eggs. To determine brood sizes, individual L4 animals from each condition were transferred onto separate plates, monitored daily during fertile egg laying and then transferred onto fresh plates to keep them separate from their progeny. Plates with eggs were incubated for an additional 48 h to allow hatching, and the number of larvae was counted. This continued until adult animals no longer laid fertile eggs.

### Quantitative PCR with reverese transcription

Total RNA was extracted with TRIzol reagent (Invitrogen) from frozen day 2 adults and reverse transcribed into single-stranded complementary DNA using QuantiTect Reverse Transcription Kit (QIAGEN) or ReverTra Ace qPCR RT master Mix with gDNA remover (TOYOBO) according to manufacturers' protocols. Quantitative PCR with reverese transcription was performed with an ABI 7300 Real-Time PCR system (Applied Biosystem) using FastStart Universal SYBR Green Master (Rox) (Roche) or SYBR Premix Ex Taq II(TaKaRa). The relative messenger RNA levels were normalized to those of *sgo-1*or *act-1*. Primer sequences are available on request.

### Western blotting

Stressed P0 parents (at day 2 adulthood) and the unstressed F1 descendants (at day 1 adulthood) were washed by M9 buffer, and frozen in liquid nitrogen. Worm extracts were lysed in sample buffer (2% SDS, 10% glycerol, 5% β-mercaptoethanol, 62.5 mM Tris-HCl pH 6.8, 0.001% bromophenol blue), followed by sonication. After centrifugation, supernatants were collected and boiled for 10 min. Samples were resolved by SDS–polyacrylamide gel electrophoresis (14% polyacrylamide gels) and subjected to immunoblotting according to a standard protocol using primary antibodies (anti-Histone H3 (Abcam ab1791, 1:1,000), anti-Histone H3K4me3 (Abcam ab8580, 1:1,000) and an anti-rabbit secondary antibody (GE Healthcare NA9340, 1:10,000)). Signals were detected with Western Lightning Plus-ECL Enhanced Chemiluminescence reagent (PerkinElmer). Band intensities were measured using ImageJ software. All uncropped western blots can be found in Supplementary Fig. 8.

### Statistics

Statistical analysis and sample sizes for each experiment are described in figure legends and Supplementary Tables. The statistical analysis was two tailed, and was performed using Excel (Microsoft) or GraphPad Prism 6.0 (GraphPad Software, Inc.). Eggs were sorted randomly to each experimental and control group. Animals that crawled off the plate, displayed extruded internal organs, or died from internally hatched progeny were censored and excluded from the statistical analysis. For multiple comparisons, Bonferroni correction was used to adjust the *P* values.

### Data availability

The authors declare that all data supporting the findings of this study are available within the article and its Supplementary Information Files or from the corresponding author on reasonable request.

## Additional information

**How to cite this article:** Kishimoto, S. *et al*. Environmental stresses induce transgenerationally inheritable survival advantages via germline-to-soma communication in *Caenorhabditis elegans*. *Nat. Commun.* **8,** 14031 doi: 10.1038/ncomms14031 (2017).

**Publisher's note:** Springer Nature remains neutral with regard to jurisdictional claims in published maps and institutional affiliations.

## Supplementary Material

Supplementary Information Supplementary Figures, Supplementary Tables.
