# Supplementary material for: Environmental stresses induce transgenerationally inheritable survival advantages via germline-to-soma communication in Caenorhabditis elegans
Source: Nat Commun. 2017 Jan 9;8:14031. doi: 10.1038/ncomms14031 (PMC5227915; doi:10.1038/ncomms14031)
Supplement: Supplementary Information — Supplementary Figures, Supplementary Tables. [file ncomms14031-s1.pdf]

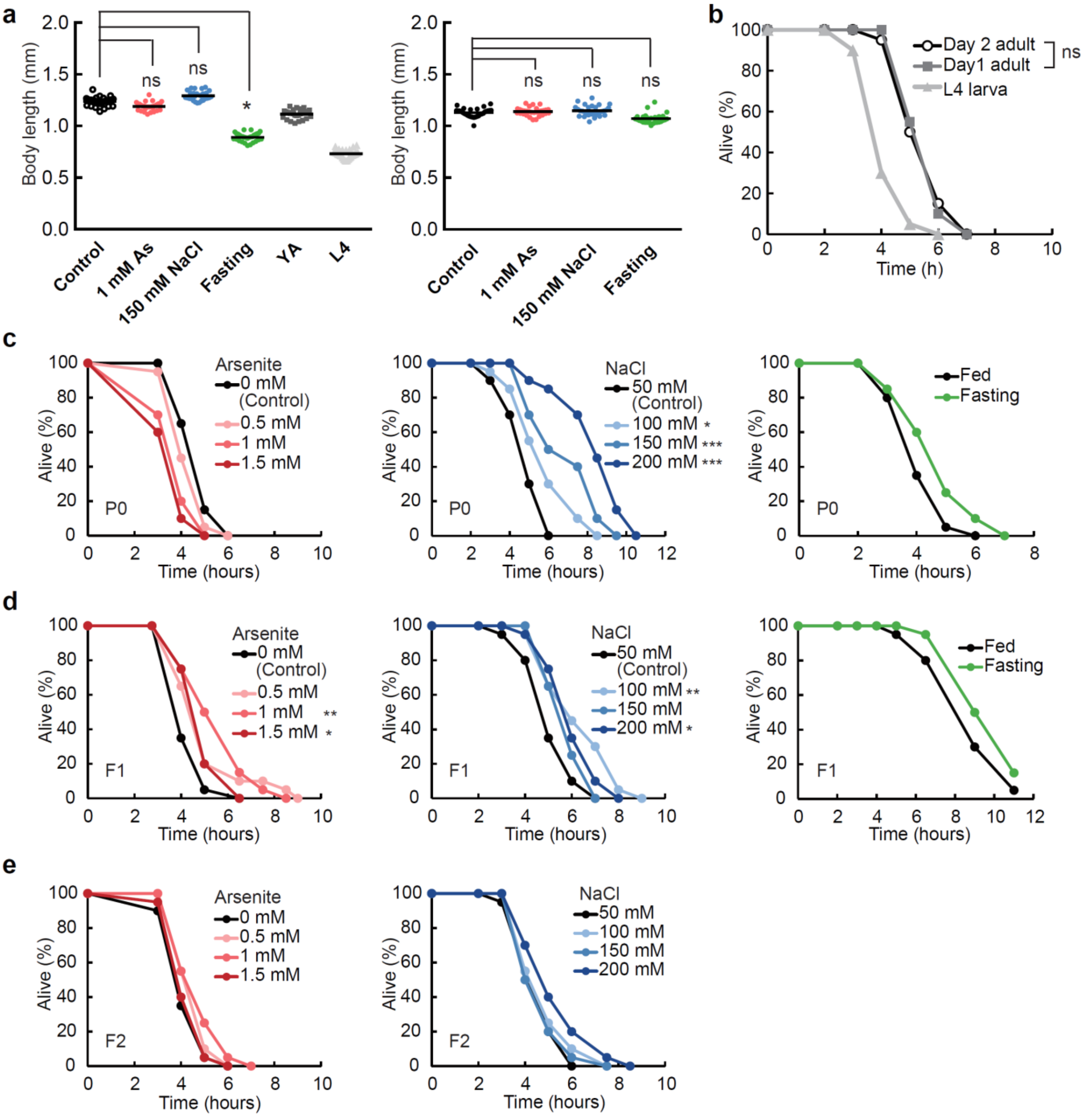

Supplementary Figure 1 Effects of stress exposure during developmental stages in the P0 generation. (**a**) Body length of the stressed P0 parents on day 2 adulthood (left), and the unstressed F1 descendants derived from the stressed P0 parents on day 1 adulthood (right). Representative data of three independent experiments are shown (n = 30). Bars represent the mean. **P* < 0.05, Student's *t*-test. (**b**) Oxidative stress resistance of day 1 adulthood (young adult), day 2 adulthood, and L4 larva in wild-type animals. There was no significant difference in stress resistance between day 1 and day 2 adulthood. Representative data of three independent experiments are shown (n = 30). log-rank test. (**c-e**) Oxidative stress resistance (250 mM paraquat) of the stressed P0 parents (**c**), self-fertilized F1 (**d**) and F2 (**e**) descendants compared to control groups. Representative data of two or three independent experiments are shown (n = 20). ns, not significant; **P* < 0.05, ***P* < 0.01, ****P* < 0.005, log-rank test (with Bonferroni correction).

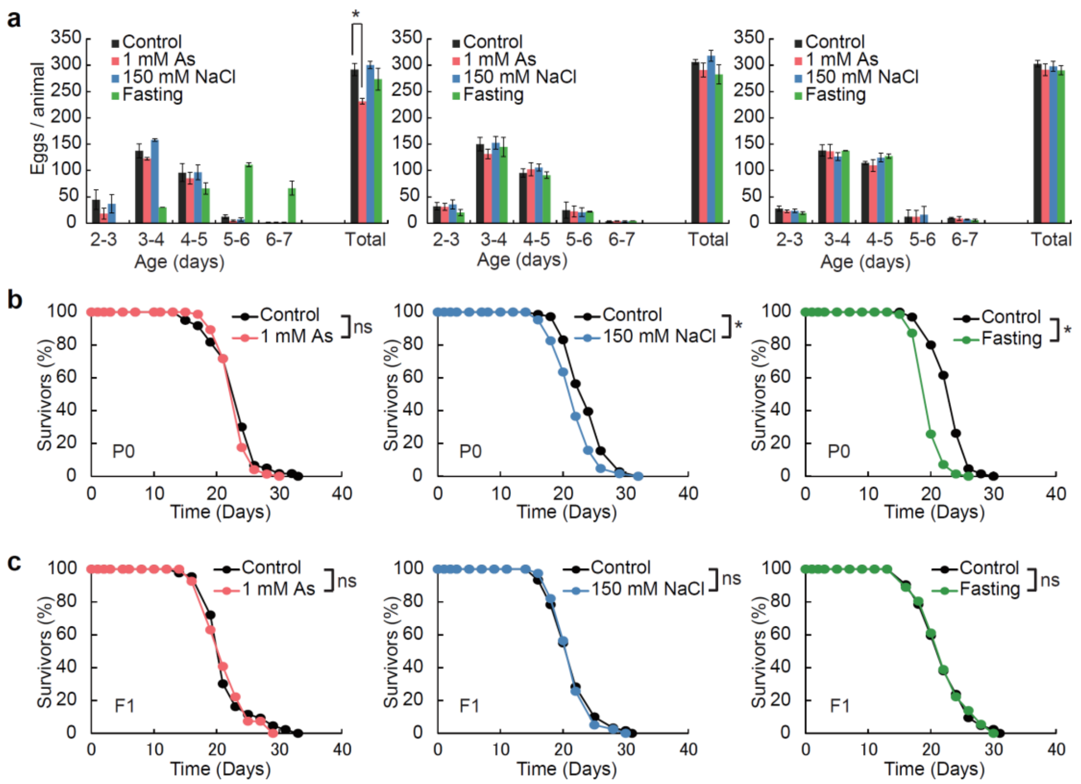

Supplementary Figure 2 Effects of stress exposure in the P0 generation on the fecundity and lifespan of the P0 and subsequent generations. (**a**) Brood size of the stressed P0 parents (left), the self-fertilized F1 (middle) and F2 (right) descendants (n = 10). Error bars represent the mean ± s.e.m. of three independent experiments, $*P < 0.05$, Student's *t*-test. (**b**, **c**) Lifespan of the stressed P0 parents (**b**) and the self-fertilized F1 descendants (**c**). P0 parents subjected to stressors exhibited a bit shortened lifespan compared to the control groups, but the F1 descendants did not show shortened lifespan. Representative data of three independent experiments are shown. ns, not significant; $*P < 0.05$, log-rank test.

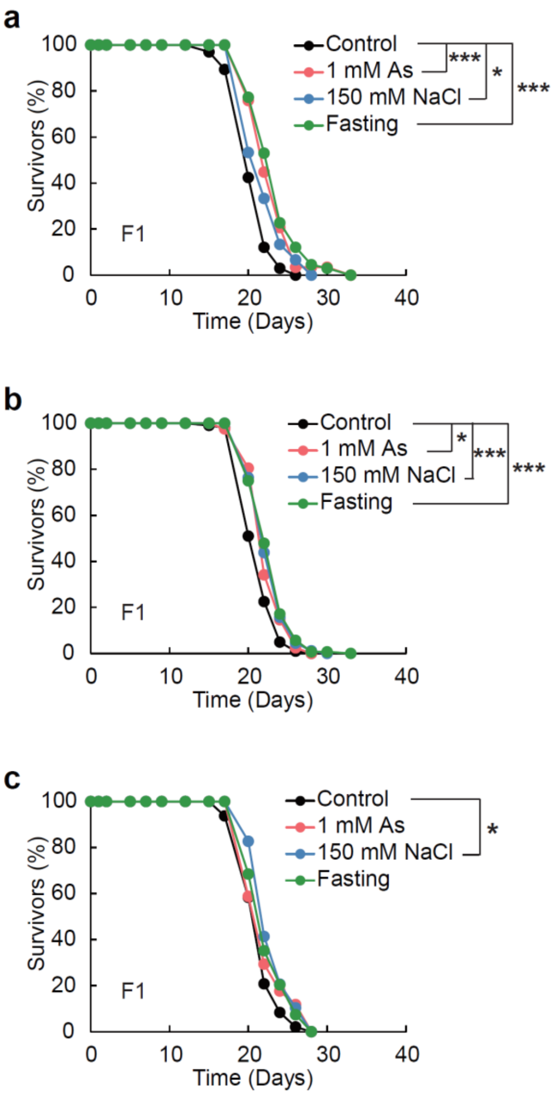

Supplementary Figure 3 Stress exposure of several transgenic male parents leads to the extended lifespan in the crossed F1 descendants. (**a-c**) Lifespan of the crossed the F1 descendants whose male parents (transgenic animals expressing P*hsp-6::gfp* (**a**), P*hsp-60::gfp* (**b**) and P*hsp-4::gfp* (**c**)) were subjected to stressors. *P < 0.05, ***P < 0.005, log-rank test (with Bonferroni correction).

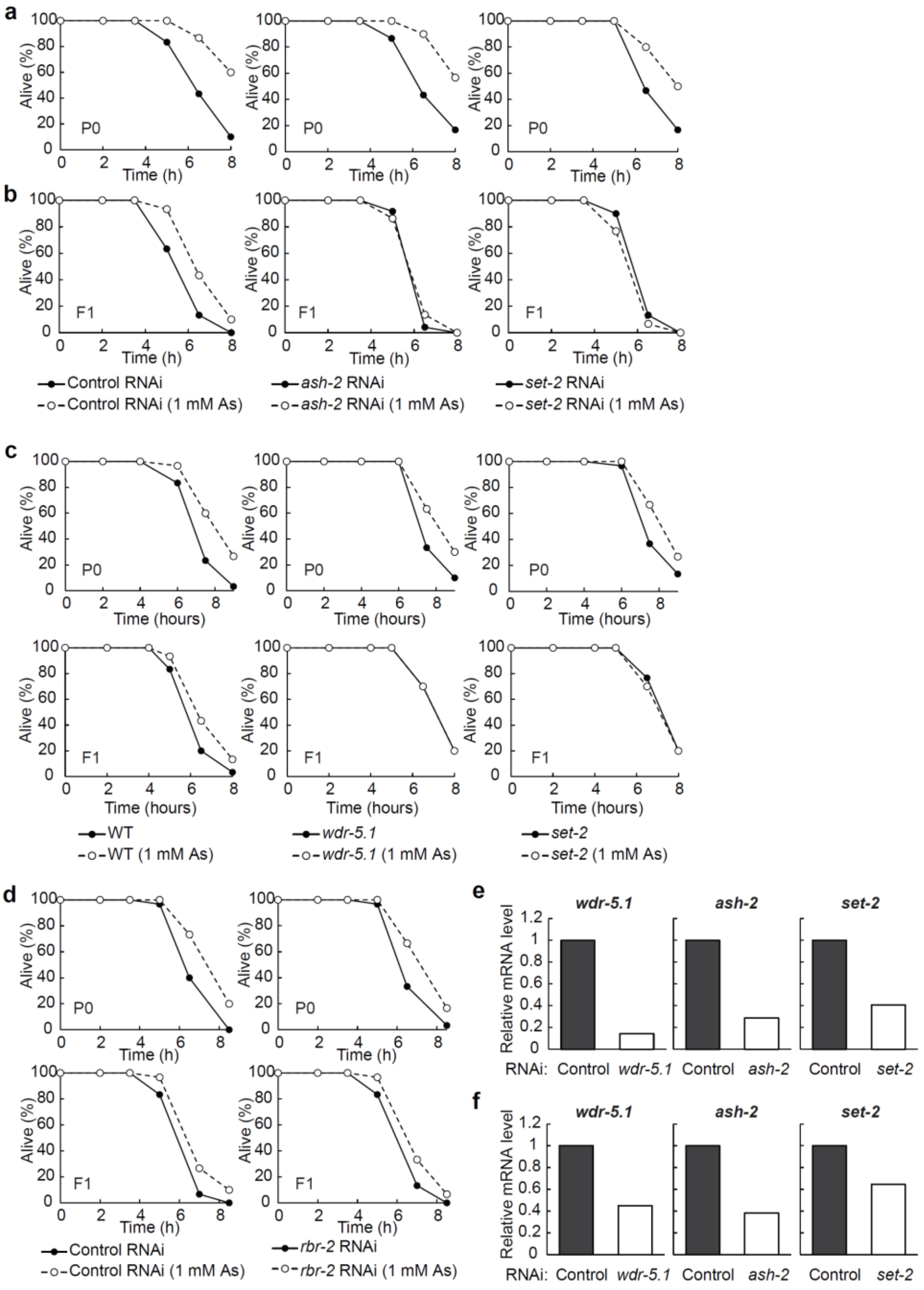

Supplementary Figure 4 The components of the H3K4me3 complex, *ash-2* and *set-2*, but not H3K4me3 demethylase *rbr-2*, are required for the transgenerational inheritance of hormesis effects. (**a**, **b**) Oxidative stress resistance (1.7 mM H_2O_2) of the stressed P0 parents treated with RNAi (left, empty vector (control); middle, *ash-2*; right, *set-2*) on day 2 adulthood (**a**) and the self-fertilized F1 descendants on day 1 adulthood (**b**) compared to control groups. Representative data of three independent experiments are shown (n = 30). (**c**) Mutation of *wdr-5.1 (ok1417)* or *set-2 (ok952)* does not suppress the increase in the stress resistance in the P0 parents (upper graphs), but does suppress it in the F1 descendants (lower graphs) on day 2 adulthood compared to control groups (left, wild type N2; middle, *wdr-5.1 (ok1417)*; right, *set-2 (ok952)*). Representative data of more than three independent experiments are shown (n = 30). (**d**) *rbr-2* RNAi does not affect the increase in the resistance to 1.7 mM H_2O_2 of either P0 parents (upper) or the F1descendants (lower). Representative data of two independent experiments are shown (n = 30). (**e, f**) Relative mRNA expression levels of *wdr-5.1*, *ash-2* and *set-2* in the stressed P0 parents (**e**) and in the self-fertilized F1 descendants (**f**). Knockdown efficiency induced by feeding RNAi persisted beyond generations. Similar results were obtained in two independent experiments.

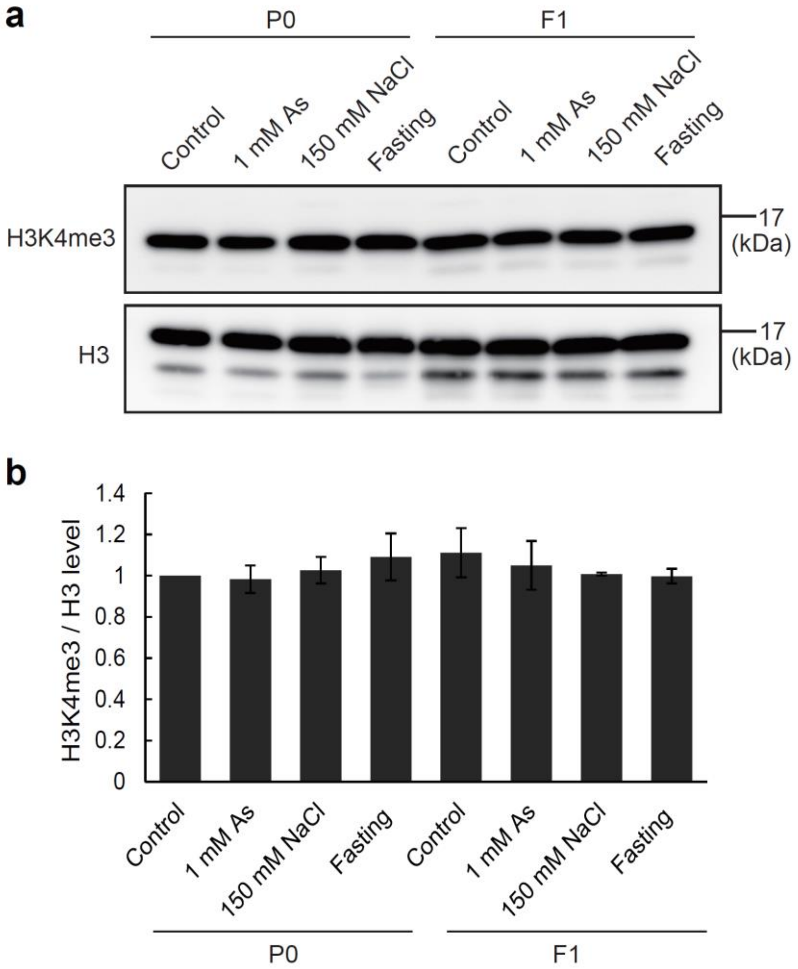

Supplementary Figure 5 No significant changes in H3K4me3 levels were observed in both stressed parents and the descendants. (**a**) Histone H3K4trimathylation levels in the stressed P0 parents (day 2 adulthood) and the unstressed F1 descendants (day 1 adulthood) as measured by Western blotting. Representative data of five independent experiments is shown. Uncropped scans are presented in Supplementary Fig. 8. (**b**) Quantification of H3K4me3 levels normalized to Histone H3 levels. Statistical significance was calculated by Student's *t*-test. Error bars represent the mean ± s.d. of five independent experiments. No statistical significance was observed in each sample compared to the P0 control.

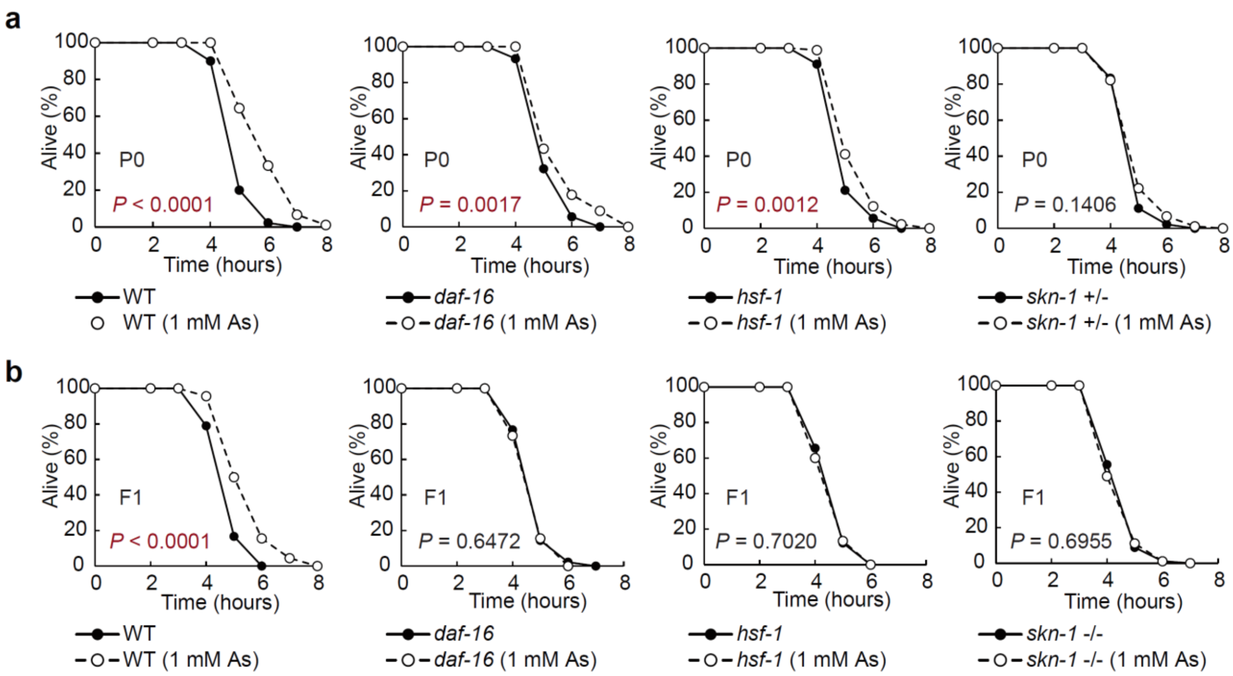

Supplementary Figure 6 Effects of mutation of *daf-16*, *hsf-1*, or *skn-1* on the transgenerational inheritance of hormesis effects. (**a**, **b**) Oxidative stress resistance (2 mM H_2O_2) of the stressed P0 parents bearing the indicated gene mutation on day 2 adulthood (**a**) and the self-fertilized F1 descendants on day 1 adulthood (**b**). Three independent experiments are integrated into each survival curve (n = 90). *P* values were calculated by log-rank test. Because the homozygotes of *skn-1* mutant show sterility, parental heterozygotes were subjected to 1 mM As for the experiments to obtain the descendants. Homozygotes (the descendants) were distinguished from heterozygotes by GFP expression. In P0 parents, *skn-1* heterozygotes were used.

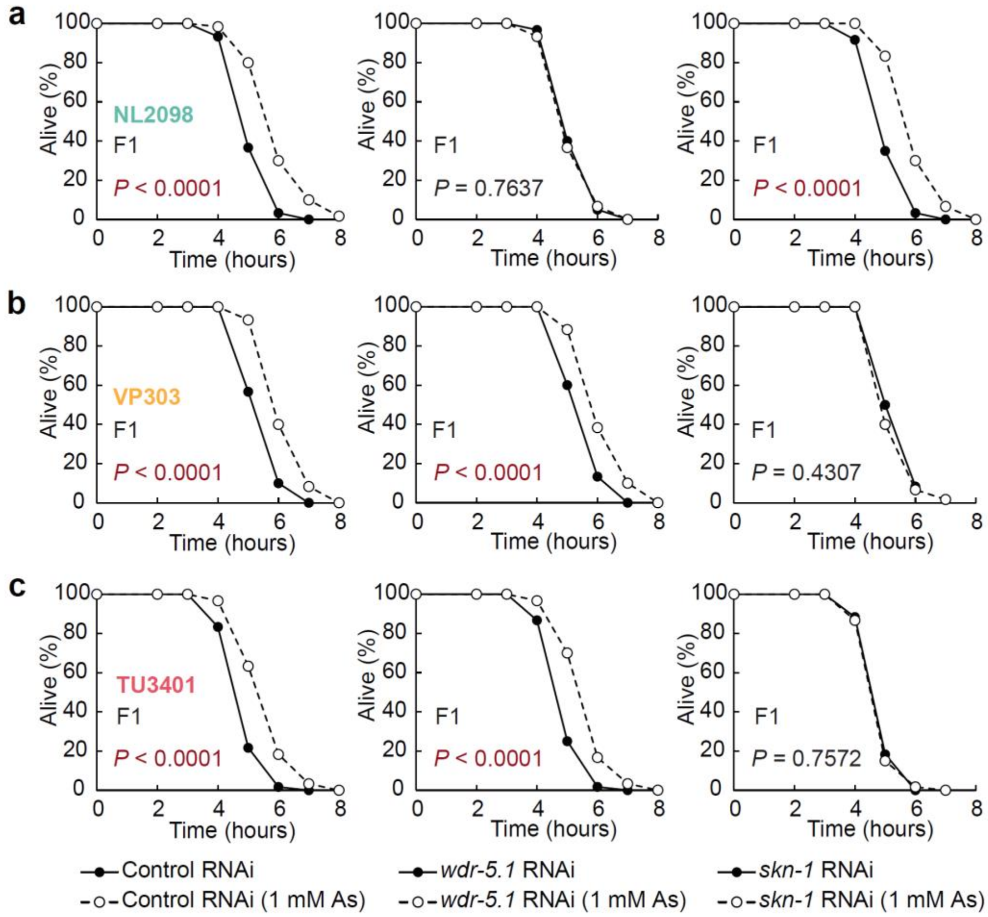

Supplementary Figure 7 Effects of tissue-specific RNAi treatment on the stress resistance of the F1 descendants. (**a**-**c**) Oxidative stress resistance (2 mM H_2O_2) of the F1 descendants on day 1 adulthood, which were derived from the stressed P0 parents and treated with RNAi (left, empty vector (control); middle, *wdr-5.1*; right, *skn-1*). Germline-specific RNAi (**a**), intestine-specific RNAi (**b**) and neuron-specific RNAi (**c**) treatments were performed in the F1 descendants, respectively. Two independent experiments are integrated into each survival curve (n = 60). *P* values were calculated by log-rank test.

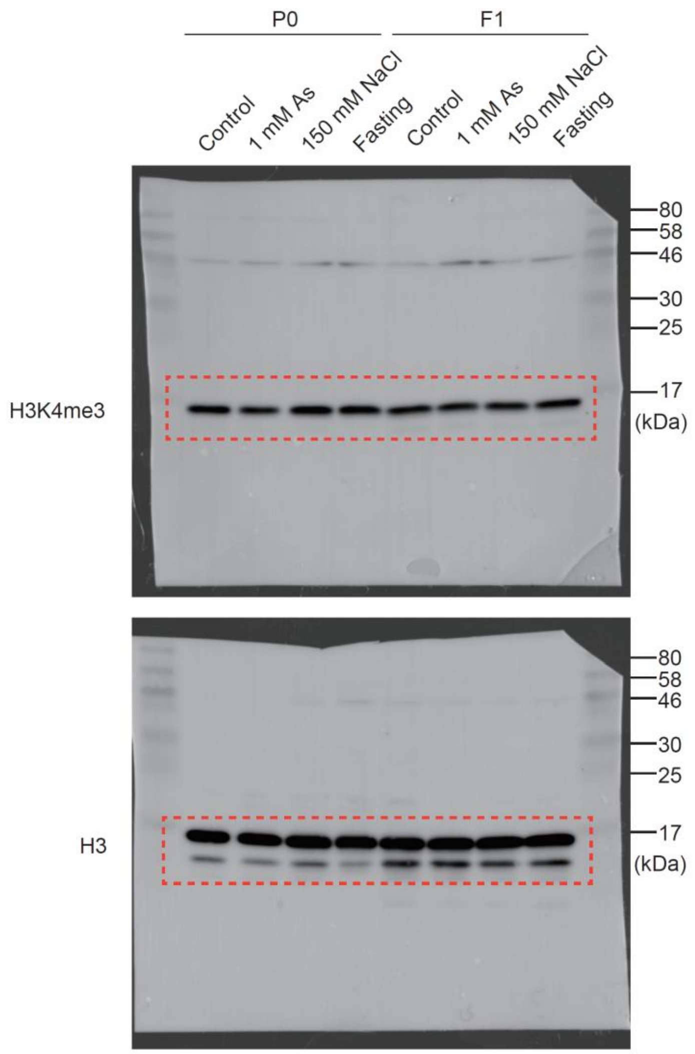

Supplementary Figure 8 Uncropped scans of blots presented in Supplementary Fig. 5.

Supplementary Table 1　Summary of stress resistance experiments

Generation	Stressors	Median survival time (hrs)	Mean survival time (hrs)	± s.d. (hrs)	% extension by stressors	# animals (N)	*P* value
**presented in Figure 1**							
P0	Control	5.5	5.44	1.17	-	80 (4)	-
	Arsenite 0.5 mM	6	6.13	1.07	12.64	80 (4)	0.0018
	Arsenite 1 mM	6	6.51	1.36	19.77	80 (4)	< 0.0001
	Arsenite 1.5 mM	6	5.90	1.43	8.51	60 (3)	0.03
	Control	6	5.82	0.95	-	60 (3)	-
	NaCl 100 mM	6	6.00	0.99	3.15	60 (3)	0.8529
	NaCl 150 mM	7	6.68	1.13	14.90	60 (3)	< 0.0001
	NaCl 200 mM	7	6.95	0.98	19.48	60 (3)	< 0.0001
	Fasting (L4)	6	6.43	1.01	10.60	60 (3)	0.0015
F1	Control	6	5.82	1.00	-	60 (3)	-
	Arsenite 0.5 mM	7	6.65	1.25	14.33	60 (3)	0.0003
	Arsenite 1 mM	7	7.07	1.42	21.49	60 (3)	< 0.0001
	Arsenite 1.5 mM	7	6.67	1.00	14.61	60 (3)	< 0.0001
	NaCl 100 mM	6	6.40	1.14	10.03	60 (3)	0.0084
	NaCl 150 mM	7	6.87	1.20	18.05	60 (3)	< 0.0001
	NaCl 200 mM	6	6.55	1.05	12.61	60 (3)	0.0009
	Fasting (L4)	6	6.69	1.27	14.97	80 (3)	< 0.0001
F2	Control	6	6.26	0.71	-	170 (5)	-
	Arsenite 1 mM	6	6.68	0.94	6.67	170 (5)	< 0.0001
	NaCl 150 mM	7	6.74	0.93	7.71	170 (5)	< 0.0001
	Fasting (L4)	7	6.78	0.98	8.36	170 (5)	< 0.0001
F3	Control	6	6.21	0.99	-	90 (3)	-
	Arsenite 1 mM	6	6.37	1.18	2.50	90 (3)	0.2791
	NaCl 150 mM	6	6.28	1.02	1.07	90 (3)	0.6492
	Fasting (L4)	6	6.49	1.09	4.47	90 (3)	0.0769
**presented in Figure 3**							
F1 descendants derived from stressed male parents							
	Control	6	6.42	0.81	-	90 (3)	-
	Arsenite 1 mM	7	7.14	1.04	11.25	90 (3)	< 0.0001
	NaCl 150 mM	7	7.12	1.06	10.90	90 (3)	< 0.0001
	Fasting (L4)	7	6.98	0.98	8.65	90 (3)	< 0.0001
F1 descendants derived from stressed hermaphrodite parents							
	Control	6	6.13	0.84	-	90 (3)	-
	Arsenite 1 mM	6	6.53	0.85	6.52	90 (3)	0.0028
	NaCl 150 mM	7	6.72	0.97	9.60	90 (3)	< 0.0001
	Fasting (L4)	7	6.63	0.83	8.15	90 (3)	0.0002

These data represent mergers of independent trials. *P* values were calculated by log-rank test (Bonferroni correction for multiple comparisons).

s.d., standard deviation; #, number of animals observed; N, number of independent trials.

Supplementary Table 2 Summary of paralysis experiments presented in Figure 2

Generation	Stressors	Mean (days)	± s.d. (days)	% extension by stressors	# animals	*P* value
P0	Control	7.89	1.56	-	99	-
	Arsenite 1 mM	9.12	2.26	15.61	108	< 0.0001
	NaCl 150 mM	9.38	2.41	18.94	94	< 0.0001
	Fasting (L4)	8.57	2.17	8.67	124	0.0255
F1	Control	6.96	1.29	-	100	-
	Arsenite 1 mM	8.16	1.89	17.26	99	< 0.0001
	NaCl 150 mM	7.16	1.60	2.80	97	0.9849
	Fasting (L4)	8.02	2.05	15.23	99	< 0.0001

P values were calculated by log-rank test with Bonferroni correction.

s.d., standard deviation; #, number of animals observed.

Supplementary Table 3 Summary of lifespan experiments presented in Figure 3

trial	Stressors	Mean lifespan (days)	±s.d. (days)	% extension by stressors	# animals	*P* value
**F1 descendants derived from stressed male parents**						
#1	Control	30.52	4.91	-	58	-
	Arsenite 1 mM	28.80	4.99	16.71	54	0.0003
	NaCl 150 mM	27.35	4.72	10.84	52	0.0245
	Fasting (L4)	24.67	5.60	23.70	50	< 0.0001
#2	Control	21.41	3.53	-	64	-
	Arsenite 1 mM	22.48	4.10	5.03	31	0.2087
	NaCl 150 mM	23.53	3.74	9.92	34	0.014
	Fasting (L4)	22.71	3.79	6.08	41	0.1112
#3	Control	25.18	6.15	-	38	-
	Arsenite 1 mM	28.03	5.95	11.29	35	0.0462
	NaCl 150 mM	27.34	5.34	8.57	35	0.1659
	Fasting (L4)	29.94	4.48	18.88	33	0.004
#4	Control	19.34	2.69	-	35	-
	Arsenite 1 mM	21.80	3.06	12.70	65	0.0001
	NaCl 150 mM	21.76	3.20	12.49	62	0.0002
	Fasting (L4)	23.02	3.35	18.99	59	< 0.0001
#5	Control	22.92	3.80	-	71	-
	Arsenite 1 mM	24.68	4.16	7.71	60	0.0169
	NaCl 150 mM	23.17	3.70	1.11	82	0.7461
	Fasting (L4)	24.86	4.04	8.46	69	0.0056
#6	Control	20.44	2.98	-	41	-
	Arsenite 1 mM	22.84	4.14	11.74	37	0.002
	NaCl 150 mM	21.20	3.00	3.72	25	0.4565
	Fasting (L4)	22.60	3.62	10.57	20	0.029
#7	Control	20.15	3.32	-	124	-
	Arsenite 1 mM	22.22	2.94	10.32	98	< 0.0001
	NaCl 150 mM	21.60	3.27	7.20	99	0.0031
	Fasting (L4)	21.80	3.22	8.21	70	0.0031

P values were calculated by log-rank test.

s.d., standard deviation; #, number of animals observed.

Exposure of male parents to environmental stresses generally led to lifespan extension in the F1 descendants. In the case of osmotic stress experiments, however, the lifespan extension was not statistically significant in three experiments out of seven.

Supplementary Table 4 Summary of stress resistance by gene knockdown experiments

Figure	Generation		Stressors	Median survival time (hrs)	# animals (N)	*P* value
4a	P0	Control RNAi	Control	6	60 (2)	-
			Arsenite 1 mM	7	60 (2)	< 0.0001
		utx-1 RNAi	Control	6	60 (2)	-
			Arsenite 1 mM	7	60 (2)	< 0.0001
		wdr-5.1 RNAi	Control	6	60 (2)	-
			Arsenite 1 mM	7	60 (2)	< 0.0001
4b	F1	Control RNAi (P0)	Control	6	60 (2)	-
			Arsenite 1 mM	7	60 (2)	< 0.0001
		utx-1 RNAi (P0)	Control	6	60 (2)	-
			Arsenite 1 mM	7	60 (2)	< 0.0001
		wdr-5.1 RNAi (P0)	Control	6	60 (2)	-
			Arsenite 1 mM	6	60 (2)	0.5113
4c	F1	Control RNAi (F1)	Control	7	90 (3)	-
			Arsenite 1 mM	7	90 (3)	< 0.0001
		wdr-5.1 RNAi (F1)	Control	7	90 (3)	-
			Arsenite 1 mM	7	90 (3)	0.3033
4d	P0	Control RNAi	Control	6	60 (2)	-
			Arsenite 1 mM	7	60 (2)	< 0.0001
		daf-16 RNAi	Control	6	60 (2)	-
			Arsenite 1 mM	7	60 (2)	< 0.0001
		hsf-1 RNAi	Control	6	60 (2)	-
			Arsenite 1 mM	7	60 (2)	< 0.0001
		skn-1 RNAi	Control	6	60 (2)	-
			Arsenite 1 mM	6	60 (2)	0.2893
4e	F1	Control RNAi (P0)	Control	6	60 (2)	-
			Arsenite 1 mM	6.5	60 (2)	< 0.0001
		daf-16 RNAi (P0)	Control	6	60 (2)	-
			Arsenite 1 mM	6	60 (2)	0.8432
		hsf-1 RNAi (P0)	Control	6	60 (2)	-
			Arsenite 1 mM	6	60 (2)	0.6049
4f	F1	Control RNAi (F1)	Control	6	60 (2)	-
			Arsenite 1 mM	7	60 (2)	< 0.0001
		daf-16 RNAi (F1)	Control	6	60 (2)	-
			Arsenite 1 mM	7	60 (2)	< 0.0001
		hsf-1 RNAi (F1)	Control	6	60 (2)	-
			Arsenite 1 mM	7	60 (2)	< 0.0001
		skn-1 RNAi (F1)	Control	6	60 (2)	-
			Arsenite 1 mM	6	60 (2)	0.6881
5b	P0	Control RNAi	Control	6	60 (2)	-
			Arsenite 1 mM	7.5	60 (2)	< 0.0001
		daf-16 RNAi	Control	6	60 (2)	-
			Arsenite 1 mM	7	60 (2)	< 0.0001
		hsf-1 RNAi	Control	6	60 (2)	-
			Arsenite 1 mM	8	60 (2)	< 0.0001
5c	F1	Control RNAi (P0)	Control	5	60 (2)	-
			Arsenite 1 mM	6	60 (2)	0.0006
		daf-16 RNAi (P0)	Control	6	60 (2)	-
			Arsenite 1 mM	6	60 (2)	0.0004
		hsf-1 RNAi (P0)	Control	6	60 (2)	-
			Arsenite 1 mM	6	60 (2)	< 0.0001
5d	P0	Control RNAi	Control	6	60 (2)	-
			Arsenite 1 mM	7	60 (2)	< 0.0001
		daf-16 RNAi	Control	6	60 (2)	-
			Arsenite 1 mM	7	60 (2)	< 0.0001
		hsf-1 RNAi	Control	6	60 (2)	-
			Arsenite 1 mM	7	60 (2)	< 0.0001
5e	F1	Control RNAi (P0)	Control	5	60 (2)	-
			Arsenite 1 mM	6	60 (2)	0.0001
		daf-16 RNAi (P0)	Control	5	60 (2)	-
			Arsenite 1 mM	6	60 (2)	0.0011

		hsf-1 RNAi (P0)	Control	5	60 (2)	-
			Arsenite 1 mM	6	60 (2)	< 0.0001
5f	P0	Control RNAi	Control	6	60 (2)	-
			Arsenite 1 mM	7	60 (2)	< 0.0001
		daf-16 RNAi	Control	6	60 (2)	-
			Arsenite 1 mM	7	60 (2)	< 0.0001
		hsf-1 RNAi	Control	5	60 (2)	-
			Arsenite 1 mM	6	60 (2)	< 0.0001
5g	F1	Control RNAi (P0)	Control	5	60 (2)	-
			Arsenite 1 mM	6	60 (2)	< 0.0001
		daf-16 RNAi (P0)	Control	6	60 (2)	-
			Arsenite 1 mM	6	60 (2)	0.0041
		hsf-1 RNAi (P0)	Control	5	60 (2)	-
			Arsenite 1 mM	6	60 (2)	< 0.0001
6a	P0	Control RNAi	Control	6	60 (2)	-
			Arsenite 1 mM	7	60 (2)	< 0.0001
		wdr-5.1 RNAi	Control	6	60 (2)	-
			Arsenite 1 mM	7	60 (2)	< 0.0001
		skn-1 RNAi	Control	5.5	60 (2)	-
			Arsenite 1 mM	7	60 (2)	< 0.0001
6b	F1	Control RNAi (P0)	Control	5	60 (2)	-
			Arsenite 1 mM	6	60 (2)	< 0.0001
		wdr-5.1 RNAi (P0)	Control	5	60 (2)	-
			Arsenite 1 mM	5	60 (2)	0.2591
6c	P0	Control RNAi	Control	6	60 (2)	-
			Arsenite 1 mM	7	60 (2)	< 0.0001
		wdr-5.1 RNAi	Control	6	60 (2)	-
			Arsenite 1 mM	7	60 (2)	< 0.0001
		skn-1 RNAi	Control	6	60 (2)	-
			Arsenite 1 mM	5	60 (2)	0.0002
6d	F1	Control RNAi (P0)	Control	6	60 (2)	-
			Arsenite 1 mM	6	60 (2)	< 0.0001
		wdr-5.1 RNAi (P0)	Control	6	60 (2)	-
			Arsenite 1 mM	7	60 (2)	< 0.0001
		skn-1 RNAi (P0)	Control	6	60 (2)	-
			Arsenite 1 mM	6	60 (2)	< 0.0001
6e	P0	Control RNAi	Control	5	60 (2)	-
			Arsenite 1 mM	6	60 (2)	< 0.0001
		wdr-5.1 RNAi	Control	5	60 (2)	-
			Arsenite 1 mM	6	60 (2)	< 0.0001
		skn-1 RNAi	Control	5	60 (2)	-
			Arsenite 1 mM	5	60 (2)	0.6206
6f	F1	Control RNAi (P0)	Control	5	60 (2)	-
			Arsenite 1 mM	6	60 (2)	< 0.0001
		wdr-5.1 RNAi (P0)	Control	5	60 (2)	-
			Arsenite 1 mM	6	60 (2)	< 0.0001
		skn-1 RNAi (P0)	Control	5	60 (2)	-
			Arsenite 1 mM	6	60 (2)	< 0.0001

These data represent mergers of independent trials. *P* values were calculated by log-rank test.

#, number of animals observed; N, number of independent trials.
